# Supplementary figures and images for: PIF4 and ELF3 Act Independently in Arabidopsis thaliana Thermoresponsive Flowering
Source: PLoS One. 2016 Aug 26;11(8):e0161791. doi: 10.1371/journal.pone.0161791 (PMC5001698; doi:10.1371/journal.pone.0161791)

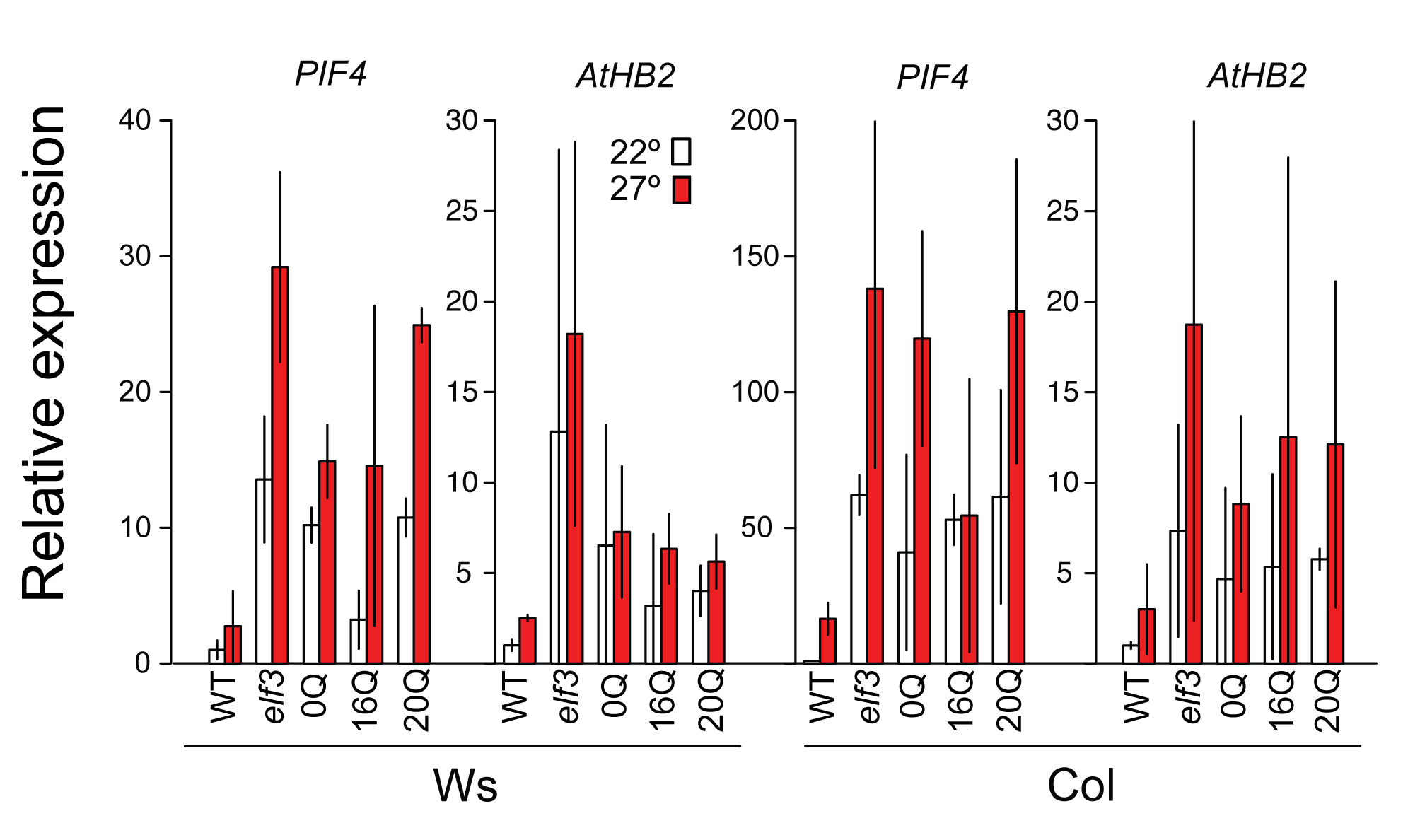

Supplement: S1 Fig — Error bars represent the standard deviation across two biological replicates. White bars represent 22° expression, red bars 27° expression for each line. Tissue was collected from 7d seedlings at ZT0. (TIF) [file pone.0161791.s001.tif]

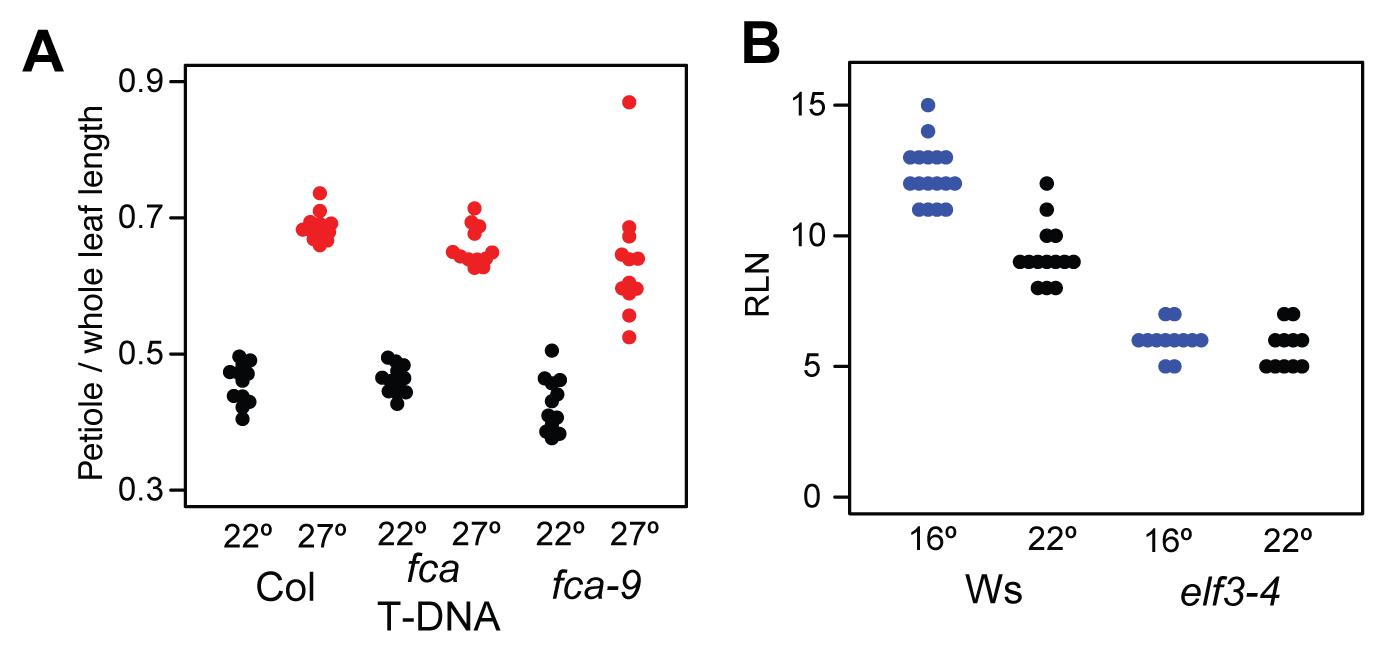

Supplement: S2 Fig — Flowering temperature response of indicated genotypes under indicated conditions, measured by petiole length to leaf length ratio at 25 days or rosette leaf number (RLN) at flowering. For each experiment, n > 10 plants for each genotype in each treatment. Outliers (defined as >1.5 interquartile ranges away from the median) of each distribution are indicated as points. Regression analysis of data in S4 and S5 Tables. (TIF) [file pone.0161791.s002.tif]

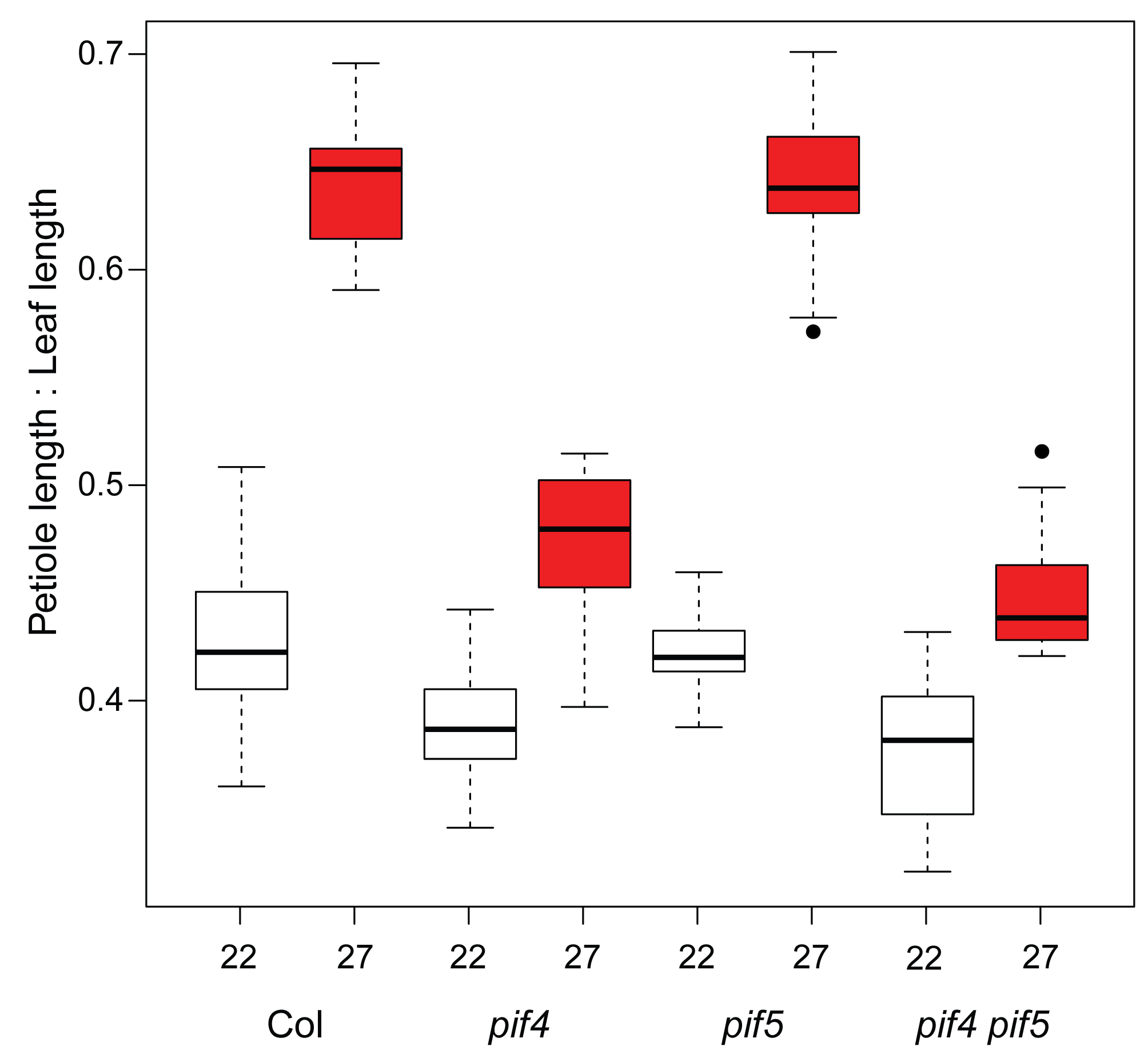

Supplement: S3 Fig — Petiole elongation temperature response of indicated genotypes under indicated conditions, measured by ratio of petiole length to leaf length at 25d. For each experiment, n > 10 plants for each genotype in each treatment. This experiment was repeated with similar results. Outliers (defined as >1.5 interquartile ranges away from the median) of each distribution are indicated as points. Regression analysis of data in S8 Table. (TIF) [file pone.0161791.s003.tif]

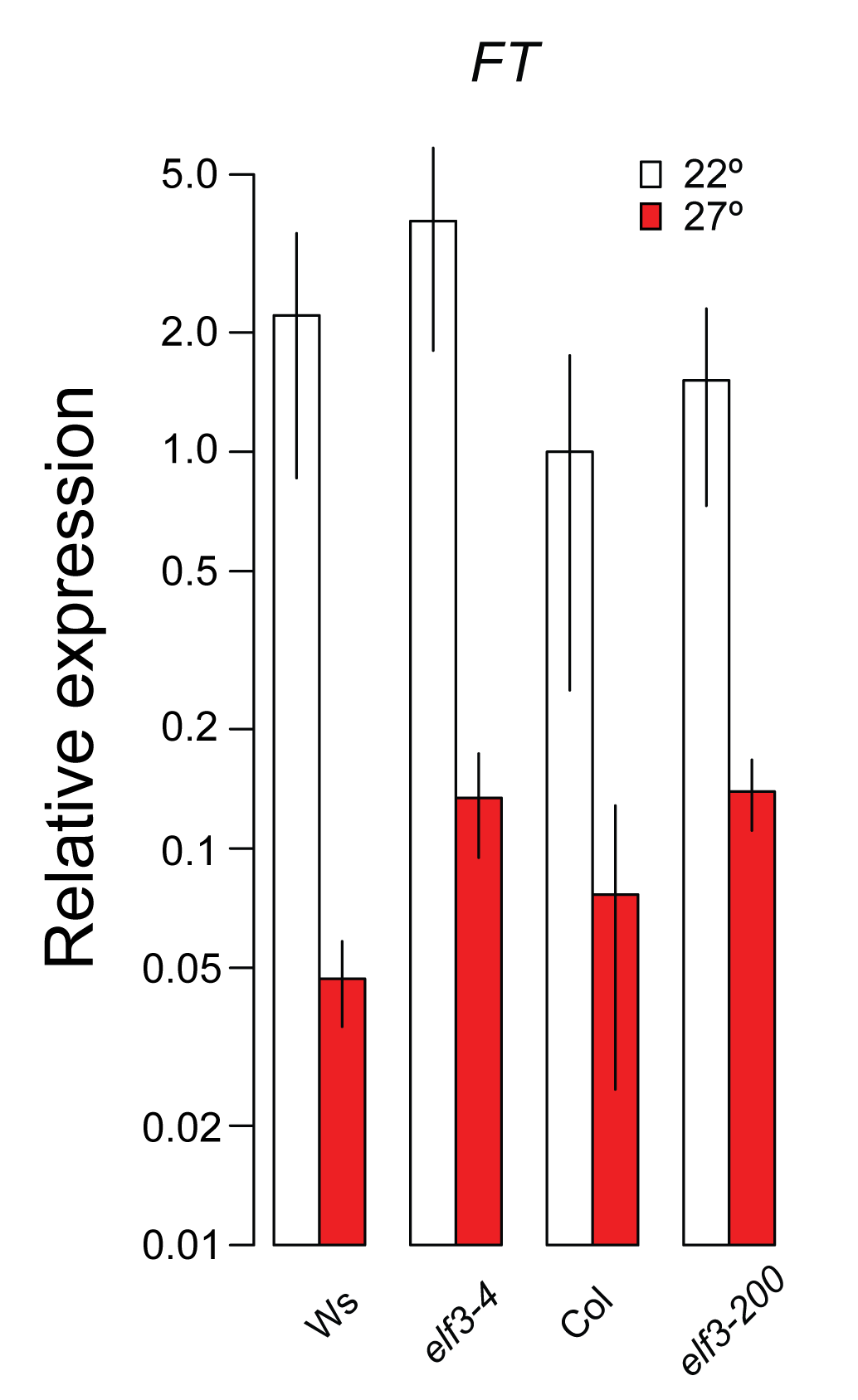

Supplement: S4 Fig — White bars represent 22° expression, red bars 27° expression for each line. Tissue was collected from 7d seedlings at ZT0. Error bars indicate SEM across three biological replicates. (TIF) [file pone.0161791.s004.tif]
